# Studying the Real-Time Interpretation of Novel Noun and Verb Meanings in Young Children

**DOI:** 10.3389/fpsyg.2019.00274

**Published:** 2019-02-18

**Authors:** Alex de Carvalho, Mireille Babineau, John C. Trueswell, Sandra R. Waxman, Anne Christophe

**Affiliations:** ^1^Laboratoire de Sciences Cognitives et Psycholinguistique, DEC-ENS/EHESS/CNRS, Ecole Normale Supérieure – PSL University, Paris, France; ^2^Maternité Port-Royal, AP-HP, Faculté de Médecine Paris Descartes, Paris, France; ^3^Department of Psychology, University of Pennsylvania, Philadelphia, PA, United States; ^4^Department of Psychology, Northwestern University, Evanston, IL, United States

**Keywords:** language acquisition, syntactic bootstrapping, language processing, noun learning, verb learning, eye movements

## Abstract

Decades of research show that children rely on the linguistic context in which novel words occur to infer their meanings. However, because learning in these studies was assessed after children had heard numerous occurrences of a novel word in informative linguistic contexts, it is impossible to determine how much exposure would be needed for a child to learn from such information. This study investigated the speed with which French 20-month-olds and 3-to-4-year-olds exploit function words to determine the syntactic category of novel words and therefore infer their meanings. In a real-time preferential looking task, participants saw two videos side-by-side on a TV-screen: one showing a person performing a novel action, and the other a person passively holding a novel object. At the same time, participants heard only three occurrences of a novel word preceded either by a determiner (e.g., “Regarde! Une dase! – “Look! A dase!”) or a pronoun (e.g., “Regarde! Elle dase!” – “Look! She’s dasing!”). 3-to-4-year-olds exploited function words to categorize novel words and infer their meanings: they looked more to the novel action in the verb condition, while participants in the noun condition looked more to the novel object. 20-month-olds, however, did not show this difference. We discuss possible reasons for why 20-month-olds may have found it difficult to infer novel word meanings in our task. Given that 20-month-olds can use function words to learn word meanings in experiments providing many repetitions, we suspect that more repetitions might be needed to observe positive effects of learning in this age range in our task. Our study establishes nevertheless that before age 4, young children become able to exploit function words to infer the meanings of unknown words as soon as they occur. This ability to interpret speech in real-time and build interpretations about novel word meanings might be extremely useful for young children to map words to their possible referents and to boost their acquisition of word meanings.

## Introduction

One of the most complex tasks that humans face during language acquisition is the acquisition of word meaning. The difficulty of this task can be best appreciated if we consider, as adults, how we feel when hearing someone talking in an unknown foreign language: we have no idea how to extract the word forms from the speech stream, and although we can observe the person who is speaking, figuring out the meanings of the words that she is producing, “on the fly,” as sentences unfold, seems impossible. How, then, can babies learn words during the first steps of language acquisition by simply listening to the sentences uttered around them? How and how efficiently, do children become able to interpret sentences in real-time as sentences unfold?

Decades of research suggest that young children can rely on the linguistic context in which the words appear (i.e., the syntactic structures of sentences) to discover the meaning of unknown words (a mechanism called *syntactic bootstrapping*, e.g., Gleitman, 1990; Gleitman et al., 2005). According to these studies, syntax can serve as a “zoom lens” allowing learners to figure out which part of the world is being talked about, which therefore helps word learners to identify candidate meanings for novel words. For instance, it has been shown that 14-month-olds are able to learn that a novel word presented as a count noun (e.g., “this one is a blicket”)” refers specifically to individual objects and categories of objects (e.g., a horse), but when that novel word appeared in an adjective form (e.g., “this one is blickish”), infants did not make such interpretation (Waxman, 1999). Around their second birthday, 24-month-olds can learn that a novel word such as “larp” refers to an event, when they listen to sentences in which this novel word appears in a verb position (e.g., “He is larping that”); but when exposed to sentences in which that novel word appeared in a noun position (e.g., “This is a larp”), toddlers interpreted “larp” as a word referring to a novel object (e.g., Bernal et al., 2007; Waxman et al., 2009; Arunachalam and Waxman, 2011, 2015; Oshima-Takane et al., 2011; similar findings were recently attested with 18-month-olds in English: He and Lidz, 2017; and in French: de Carvalho et al., 2019).

Going further, several studies demonstrated that 2-year-olds can use syntax even more specifically, not only to identify that a novel word is a verb or a noun but also to infer what kind of event a given verb is referring to depending on the syntactic structure in which it appears. For instance, 2-year-olds interpret a novel verb such as “blicking” as referring specifically to a causal event between two participants when they listen to transitive sentences such as “She is blicking the baby,” but they do not build the same interpretation about that novel verb when they listen to intransitive sentences such as “She is blicking” (e.g., Yuan and Fisher, 2009; Arunachalam and Waxman, 2010; Scott and Fisher, 2012; Yuan et al., 2012; Dautriche et al., 2014; Messenger et al., 2015; Arunachalam et al., 2016; Suzuki and Kobayashi, 2017; Arunachalam and Dennis, 2018). Moreover, recent studies demonstrated that 19- and 24-month-olds exposed to sentences like “The vep is crying” inferred that “vep” referred to an animate entity (i.e., a novel animal), because it appeared in the subject position of a familiar verb that requires an animate agent; in contrast, participants who were exposed to sentences like “The vep is right here” showed no preference for an animate entity at test (Ferguson et al., 2014, 2018).

Taken together, all these studies show that at an age when infants still don’t know the meaning of many words, they can already exploit the syntactic context of sentences to discover word meaning: they exploit the syntactic environment of a word to determine its syntactic category (e.g., as nouns, adjectives or verbs) and they use the syntactic category to restrict the kind of meaning the novel word can have (i.e., words referring to categories of objects, object properties or events). If we take a sentence processing perspective on these findings, we would like to know what kind of information children use to access the syntax of a sentence before acquiring the meaning of words, and *how* and *when* during sentence processing these interpretations about novel words are constrained.

In order to exploit the linguistic context of sentences and to figure out their syntactic structures, several studies propose that function words and morphemes (i.e., articles, pronouns, functional morphemes, case markers, etc.) and their distribution in the input could be an important and reliable source of information for young children (e.g., Shi et al., 1998, 2006; Mintz et al., 2002; Mintz, 2003; Christophe et al., 2008, 2016; Chemla et al., 2009; Weisleder and Waxman, 2010; Shi, 2014). This hypothesis is based on the fact that function words are acquired within the 1st year of life, because they are highly frequent (much more frequent than content words: nouns, verbs, adverbs), and they possess perceptual and distributional characteristics that distinguish them from content words (e.g., Shi et al., 1998, 1999). Because functional elements tend to consistently co-occur with content words from specific word categories (e.g., determiners such as “a,” “the” typically co-occur with nouns, while pronouns like “she,” “he” and “they” tend to co-occur with verbs), the idea is that infants could use the distributional information in their input to learn about function words and to identify which words or sets of words co-occur with words from specific categories (e.g., Mintz, 2003; Chemla et al., 2009; Weisleder and Waxman, 2010). In other words, during the first steps of language acquisition, young children still don’t know much about the meanings of content words in their language, but they could use function words to determine the syntactic category of the words that they don’t know yet, and this information in turn might help them to infer the possible meaning of novel words and focus their attention to what has been talked about in their environment (e.g., Christophe et al., 2008, 2016; Shi, 2014). For instance, when children listen to a sentence such as “It’s *a* dax,” they should infer that since *dax* is being used as a noun, it probably refers to a kind of object in their environment because other already learned words in this syntactic context tend to be object-denoting terms. However, when listening to a sentence such as “It’s daxing,” infants should infer that since this novel word is being used as a verb, it probably refers to a kind of action/event that is being performed by something in their environment because other already learned words in this syntactic context tend to be event-denoting terms.

Supporting this hypothesis, previous work demonstrated that child-directed speech contains distributional regularities such as functional elements and frequent frames (e.g., jointly occurring words) that can indeed support the discovery of grammatical categories such as noun and verb in infants (e.g., Mintz et al., 2002; Mintz, 2003; Chemla et al., 2009; Weisleder and Waxman, 2010). Crucially, several experimental studies have shown that infants recognize the function words in their native language during their 1st year of life (e.g., in English: Shi et al., 2006; and in French: Shi and Lepage, 2008), and between 12- and 24-months of age, infants become able to exploit function words to determine the syntactic category of subsequent content words (Bernal et al., 2007; Zangl and Fernald, 2007; Waxman et al., 2009; Shi and Melançon, 2010; Cauvet et al., 2014; Haryu and Kajikawa, 2016; He and Lidz, 2017; de Carvalho et al., 2019). For instance, after being exposed to several sentences in which a novel word such as *crale* is preceded by a determiner (i.e., “*ton crale*” – “your crale”), 14-month-olds show surprise if they hear this novel word presented in a verb context (i.e., “*tu crale*” – “you crale”), but not when they hear this novel word in another noun context (i.e., “*des crale*” – “the crale”; e.g., Shi and Melançon, 2010). What remains unclear from these studies however is whether young infants can exploit the function words in a sentence not only to determine the syntactic category of novel words, but crucially, also to constrain the possible meanings of novel words and *when* during sentence processing these interpretations are constrained. Can children rely on the information carried by function words in real-time to determine the syntactic category of the novel words and to assign meanings to them “on the fly” as sentences unfold, or do they need to hear several occurrences of a novel word in a given syntactic context before they can start building hypotheses about word meanings?

Only a few studies in the literature investigated how young children process function words in real-time as sentences unfold and the results suggest that from 12 months onward, infants might be able to rely on the information carried by function words in real-time to guide their lexical access to familiar words (Kedar et al., 2006, 2017; Cauvet et al., 2014). For instance, in Kedar et al. (2017), in a preferential looking task, 12-month-olds were exposed to both grammatical sentences using the determiner “the” (i.e., “Can you see the ball?”) and ungrammatical conditions in which “the” was replaced by another English function word or omitted (e.g., “Can you see by ball?”). The results showed that infants oriented faster to a target image (e.g., the ball) following grammatical sentences than ungrammatical sentences. In Cauvet et al. (2014), 18-month-olds who were taught to recognize (and turn their heads when they listened to) a familiar target noun (‘*la balle*’ – ‘the ball’) were better able to identify this target word at test when it was preceded by a determiner (a noun context: ‘*j’aime les balles*’ – I love the balls) than when it was preceded by a pronoun (a verb context: ^∗^’Pierre, il balle du chocolat’ – ^∗^Pierre, he balls some chocolate) and conversely for target verbs. These findings suggest that function words facilitate lexical access to the neighboring known content words, and that they constrain lexical access of known words in real-time in children under age two. What has never been investigated however is whether young children could exploit the function words in a sentence, not only to determine the syntactic category of familiar words (or to facilitate their lexical access), but also to constrain the possible meanings of novel words in real-time as sentences unfold.

Previous studies conducted in English, French and Japanese demonstrated that the ability to exploit function words to determine the syntactic category of novel words can indeed help children around age two to constrain the meanings of novel words (e.g., Naigles, 1990, 1996; Waxman, 1999; Waxman and Booth, 2001; Bernal et al., 2007; Imai et al., 2008; Booth and Waxman, 2009; Yuan and Fisher, 2009; Arunachalam and Waxman, 2010, 2011, 2015; Oshima-Takane et al., 2011; Matsuo et al., 2012; Yuan et al., 2012; Dautriche et al., 2014, 2015; Messenger et al., 2015; Arunachalam et al., 2016; He and Lidz, 2017; Lidz et al., 2017; Arunachalam and Dennis, 2018; de Carvalho et al., 2019). However, in these studies, young children were first taught the meaning of a novel content word while listening to several repetitions of sentences, during a familiarization phase, and later were tested on their interpretation, during a test phase. Thus, little is known about whether infants can rely on the information carried by function words in real-time to determine the syntactic category of the novel words and to assign meanings to them. More importantly, since assessments of learning in these studies only occurred after children heard numerous occurrences of a novel word in informative linguistic contexts, it is impossible to determine how much exposure would be needed for a child to learn from such information and start building hypotheses about word meanings.

In all the studies investigating the acquisition of novel nouns and verbs in young children, they used adaptations of the paradigm developed by Bernal et al. (2007) and Waxman et al. (2009): participants were first familiarized with a video of an actor performing an action on an object and at the same time they heard several^1^ sentences supposed to teach them the meaning of a novel noun (i.e., referring to the object in the video) or a novel verb (i.e., referring to the action the actor was performing in the video). It was only after the familiarization phase that participants were tested on their understanding of the meaning of the novel words, during a test phase. For instance, in Waxman et al. (2009), 2-year-olds were first familiarized with a video showing a man waving a balloon. At the same time, participants heard several sentences presenting a novel word as either a verb (e.g., “Look! The man is *larping* a balloon – Yay! He is *larping* that!”) or as a noun (“Look! The man is waving a *larp*! Yay! That is a *larp*!”). A few seconds later, children were exposed to a test trial in which they saw two scenes side-by-side on a TV-screen: one video showing the familiarized action and object (e.g., a man waving a balloon) and the other video showing a novel action being performed on the same familiar object (e.g., a man tapping a balloon). Participants were then prompted to look at “*which one is he larping?*” (verb test) or at “*which one is a larp?*” (noun test). The results showed that participants familiarized with the verb sentences learned that “larp” referred to the waving action and thus during the verb test they looked more to the video where the man was waving. In contrast, participants exposed to noun sentences learned that “larp” referred to an object, and thus since there was a balloon in both videos, during the noun test they looked equally long to both videos. The same pattern of results was observed in French even when the novel words were preceded only by function words, such as “It is a *poune*!” for noun sentences or “It is *pooning*” for verb sentences (Bernal et al., 2007). Although these prior studies are very informative and show that young children can use the linguistic context in which a novel word is presented to make inferences about a novel noun and verb meaning, we still don’t know how much exposure is needed for a child to exploit function words to categorize novel words, and, especially, at which point in exposure young children start making inferences about novel word meanings based on the information carried by function words.

It is now well established that both adults and young children attempt to interpret speech “in real time,” making rapid guesses about the intended meaning of a sentence containing familiar words, as each word is encountered in the input (for reviews see, e.g., Tanenhaus and Trueswell, 2006; Trueswell and Gleitman, 2007). However, when the speech contains words that young children do not know yet, how efficiently do they exploit the linguistic context surrounding the unknown words to infer their meanings? While previous studies demonstrated that young children can use function words in real-time to constrain lexical access to known content words, we still don’t know whether children could also exploit function words in real-time to determine the syntactic category of novel words and therefore infer their meanings. Given the results we mentioned above, it is likely that young children might be able to exploit function words in real-time to make predictions about novel word meanings. However, an alternative hypothesis is that when young children encounter a novel content word, they need to hear several repetitions of that novel word in order to be able to compute the constraints that the syntactic context(s) in which it has been heard impose on its meaning.

The present work investigates whether only three occurrences of a novel word used as either a noun or as a verb would provide enough evidence for a child to exploit its syntactic context and therefore to infer its meaning. In order to assess the role played by function words in this process, we measured the speed with which young children can exploit function words to constrain their interpretation of novel nouns and verbs, by tracking learning over time, after each exposure to the novel word. When listening to a sentence in which the neighboring function words suggest that a novel word is a noun (e.g., Look! A dase!), or a verb (e.g., Look! She’s dasing!), can infants rapidly constrain their interpretation of the meaning they assign to this novel word, mapping nouns to objects and verbs to actions? Such an ability to constrain the interpretation of a novel word quickly, upon encountering it only a few times, would be extremely useful for young children, since in real-life, they may often not have access to many repetitions of the same word to guess its meaning. Being able to rely on morpho-syntactic cues to exploit the syntactic context of a novel word to infer its potential meaning would represent an extremely powerful learning mechanism for young word learners, as the syntactic structure of sentences can help them to constrain their interpretation about what aspect of the world is been talked about (e.g., Landau and Gleitman, 1985; Gleitman, 1990; Fisher et al., 1994; Gleitman et al., 2005; Medina et al., 2011).

## Experiment

**FIGURE 1:**
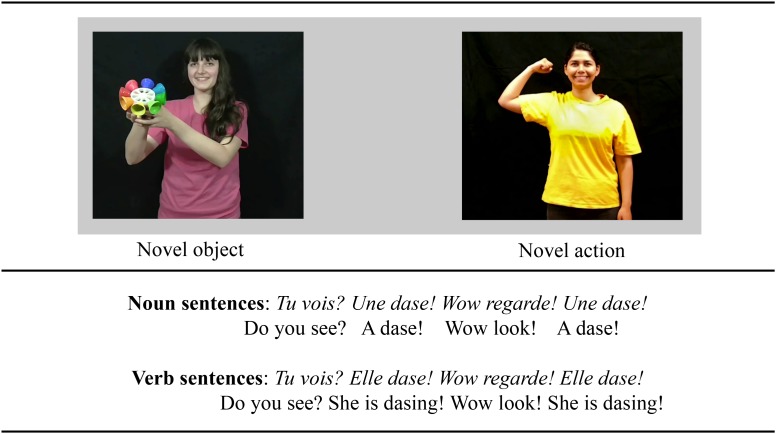
Example of the sentences and pair of videos used in the experiment. In a between-participants design, participants listened to sentences presenting a novel word either as a noun (Noun condition) or as a verb (Verb condition). At the same time, participants were presented with two videos displayed side-by-side on a TV screen, one video showing an agent performing an intransitive novel action (i.e., congruent with a verb interpretation), and the other video showing an agent simply holding a novel object (i.e., congruent with a noun interpretation).

This experiment tested whether only three occurrences of a novel word preceded either by a determiner or by a pronoun would provide enough evidence for young children to rapidly use functional elements to infer the possible meaning of novel nouns and verbs in French. Four novel words such as “dase,” “fome,” “rane,” and “nuve” were presented either as nouns or as verbs depending only on the function words that preceded them. For instance, in the sentence “*Regarde, une dase*” (“Look, a dase!), the novel word “dase” should be considered as a noun because it is preceded by a determiner, but in the sentence “*Regarde, elle dase*” (“Look, she’s das*ing*”), the novel word “dase” should be considered as a verb because it is preceded by a pronoun. As children listened to this kind of sentences, they watched two videos displayed side-by-side on a TV-Screen: one video showing a person doing a novel action, and another video showing a person holding a novel object (see Figure 1). If young children can rapidly use function words to constrain their interpretation of the novel word meanings, we expect them to look more toward the video showing a person doing a novel action when listening to sentences presenting the novel words as a verb (e.g., “*Regarde, elle dase*” – “Look, she’s dasing”) than when listening to sentences in which the novel word was presented as a noun (e.g., “*Regarde, une dase*” – “Look, a dase!”). By measuring young children’s learning behavior in real-time, this study can determine at which point during sentence processing, or from which occurrence of the novel word (from the first to the third) young children reveals signs of novel noun and verb learning.

We decided to test two groups of participants: a group of children under 2 years of age (i.e., the 20-month-old group), and another group older than 3 years of age (i.e., the 3-to-4-year-old group). The age range of participants and the expected number of participants in each condition were decided based on previous studies showing that 18-month-olds (He and Lidz, 2017; de Carvalho et al., 2019) and 24-month-olds (Bernal et al., 2007; Waxman et al., 2009) successfully learned a novel noun or a novel verb based on the function words preceding the target words. Given infants’ success at 18 months of age, we expected the ability to use function words to infer the meaning of novel words to be surely active at 20 months. However, there was no evidence in the literature that at 20-months, infants would be able to exploit function words to constrain the meanings of novel nouns and verbs in *real-time* as sentences unfold or with only three occurrences of the novel word (i.e., at the same time as they hear the sentences and they watch two dynamic scenes on a TV-Screen). Thus, we decided to also test an older group of participants (i.e., children older than 3 years of age) for whom we expected this ability would be present. Although there is no evidence in the literature showing that 3-to-4-year-olds would be able to exploit function words in real-time to determine the syntactic category of novel words and infer their meanings, there is at least evidence that 3-to-5-year-olds can succeed in tasks where they needed to discover the meaning of novel verbs while watching dynamic scenes at the screen (e.g., Imai et al., 2008; Nappa et al., 2009; Arunachalam et al., 2016) and they also succeed to learn novel word meanings after have heard only three occurrences of a novel word in informative linguistic contexts (e.g., Imai et al., 2005, 2008).

## Materials and Methods

The study reported in this paper, including the entire method, analysis and criteria for exclusion of participants were pre-registered on the OSF (Open Science Framework) database before running the experiment. The preregistration can be accessed with the following link: https://osf.io/wmnvg/?view_only=89ee189843b34f01bc81a14a86396141. The materials, collected data, and data analysis are freely available to readers through the same link.

### Participants

Ninety-seven children participated. They were all monolingual native French speakers with less than 20% exposure to another language. Participants were divided into two age groups: the 20 month-old group (*N* = 49), ranging in age from 19;5 (months; days) to 21;0, with a mean of 20;1 (*SD* = 0.27 months, 23 girls) and the 3-to-4-year-old group (*N* = 48), ranging in age from 38;6 (months; days) to 50;3, with a mean of 44;9 (*SD* = 3.10 months, 23 girls). Within each age group, participants were randomly assigned to one of the two experimental conditions: the noun or verb condition. The final sample for 20-month-olds contained 27 participants in the noun condition and 22 participants in the verb condition. The final sample for 3-to-4-year-olds contained 25 participants in the noun condition and 23 participants in the verb condition. 20-month-olds were tested in the lab; 3-to-4-year-olds were tested in a public preschool in Paris. This study was carried out in accordance with the recommendations of our local ethics committee (i.e., Comité d’éthique de la recherche en santé – CERES, Paris), with written informed consent from all parents of our participants. All parents gave written informed consent in accordance with the Declaration of Helsinki. Our protocol was also approved by the Comité d’éthique de la recherche en santé – CERES, Paris.

An additional eighteen 20-month-olds and six 3-to-4-year-olds participated but were not included in the final analysis due to fussiness during the experiment (6 toddlers, 1 preschooler), technical problems (3 preschoolers), exposure to other languages than French at home (1 toddler, 2 preschoolers), crying during the experiment (2 toddlers), or because of missing eye-tracking data representing more than 50% of unusable test trials (9 toddlers).

### Apparatus

The 20-month-olds were tested individually in a double-walled sound-attenuated booth (IAC Acoustics) in our lab. They sat on their parent’s lap, facing a 27-inch television positioned 70 cm away from them. The caregivers wore headphones and listened to masking music during the experiment. The experimenter stayed outside the booth during the test. The 3-to-4-year-olds were tested individually in a quiet room in their own preschool. They sat alone approximately 70 cm away from a 27-inch computer screen displaying the visual stimuli and they wore headphones to listen to the audio stimuli. Participants’ eye movements were recorded by an eye-tracker (Eyelink 1000) placed below the screen, and operating in a remote mode with a time-sample collected every 2 ms.

### Materials

**FIGURE 2:**
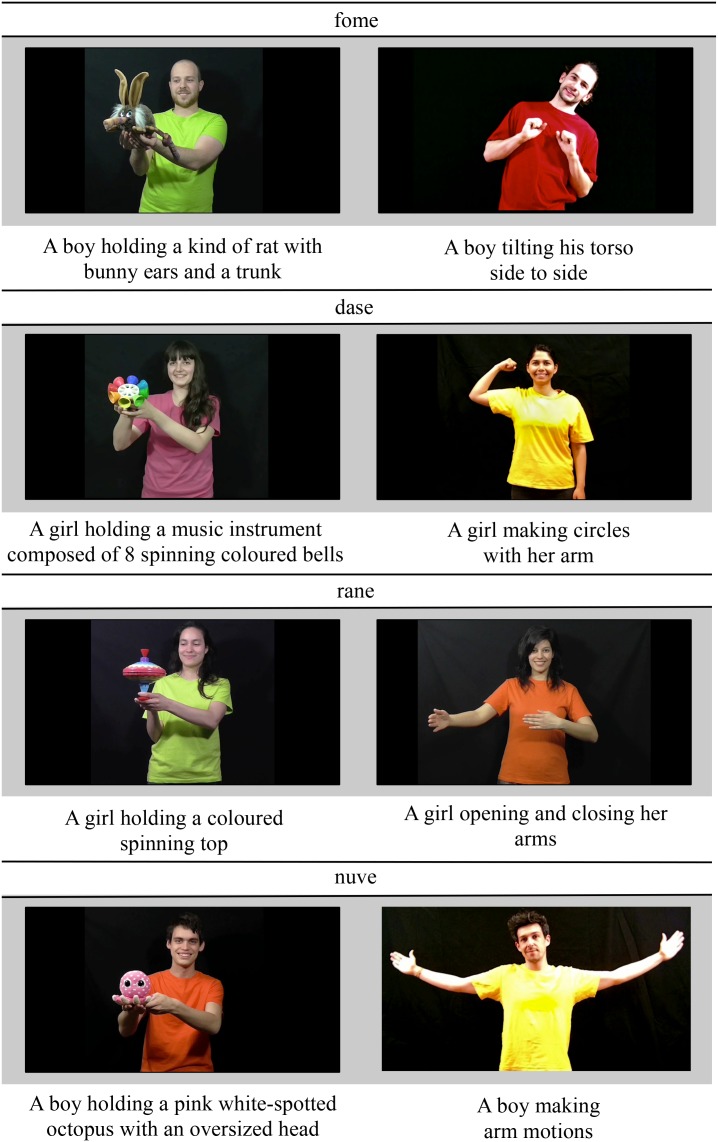
Novel words and videos used in the Experiment.

Materials consisted of four pairs of color videos showing people performing novel self-generated actions or people passively holding and looking to novel objects. All the “actors” in these videos were consenting adults who accepted to have their image used within the framework of this study (stimuli and publication of the current paper). All the actors participated on a voluntary basis, with no financial compensation. Each pair of videos was used to illustrate the possible interpretations of one of the four novel words used in the experiment: “fome,” “dase,” “rane,” and “nuve” (see Figure 2 for the description of each video).

These videos offered participants the option of interpreting the novel words either as a noun, referring to an object, which can be observed only in the video showing a person holding a novel object, or as a verb, referring to an action, which can be observed only in the video showing a person doing a novel action.

Additionally, two pairs of videos illustrating familiar words (two nouns: *une voiture, un ballon* – a car, a ball, and two verbs: *elle dort*, *il mange* – she is sleeping, he is eating) were created and used as practice trials. These videos were similar to the test videos, the only difference was that the target words were familiar for children. For instance, in one pair of videos, one video presented a girl holding a car (i.e., *une voiture*) and the other video presented a girl sleeping (i.e., *elle dort*). In the other pair of familiar videos, one video presented a boy holding a ball (i.e., *un ballon*) and the other video presented a boy eating (i.e., *il mange*).

Note that in each pair of videos, the person holding an object or the person doing an action were matched for gender. This insured that participants could not use the gender of the pronouns or the articles preceding the target words to find which video was talked about. Each actor appeared in only one video.

All videos were accompanied by sound tracks recorded by a female native French speaker (last author), who uttered all sentences in child-friendly speech. These sound tracks presented the novel words in one of the two experimental conditions (i.e., noun condition or verb condition). The sound tracks for the noun condition presented the novel target word in sentences such as “*Tu vois? Une dase! Wow regarde! Une dase!*” (“Do you see? A dase! Wow look! A dase!”), in which the target word “dase” occupied a noun position in the sentences and was preceded by a determiner. The sound tracks for the verb condition presented the novel target word in sentences such as “*Tu vois? Elle dase! Wow regarde! Elle dase!*” (“Do you see? She is dasing! Wow look! She is dasing!”), in which the target word occupied a verb position in the sentences, since it was preceded by a pronoun. Note that for each sound track, for each trial in this experiment, the target word was repeated twice.

### Procedure

The procedure included six trials: two practice trials involving a familiar word (one noun target and one verb target) common to all participants, and four novel-word test trials (“fome,” “dase,” “rane,” and “nuve”) presented in one of the two experimental conditions, in a between-participants design. Each item included a 10s test trial in which a pair of videos was presented together with the sentences. Each participant participated in 6 trials: 2 familiar trials followed by the 4 novel-word trials. Participants’ eye-gaze toward the videos was recorded by an eye-tracker during the experiment. Each experimental session began with a five-point eye-tracking calibration routine.

**FIGURE 3:**
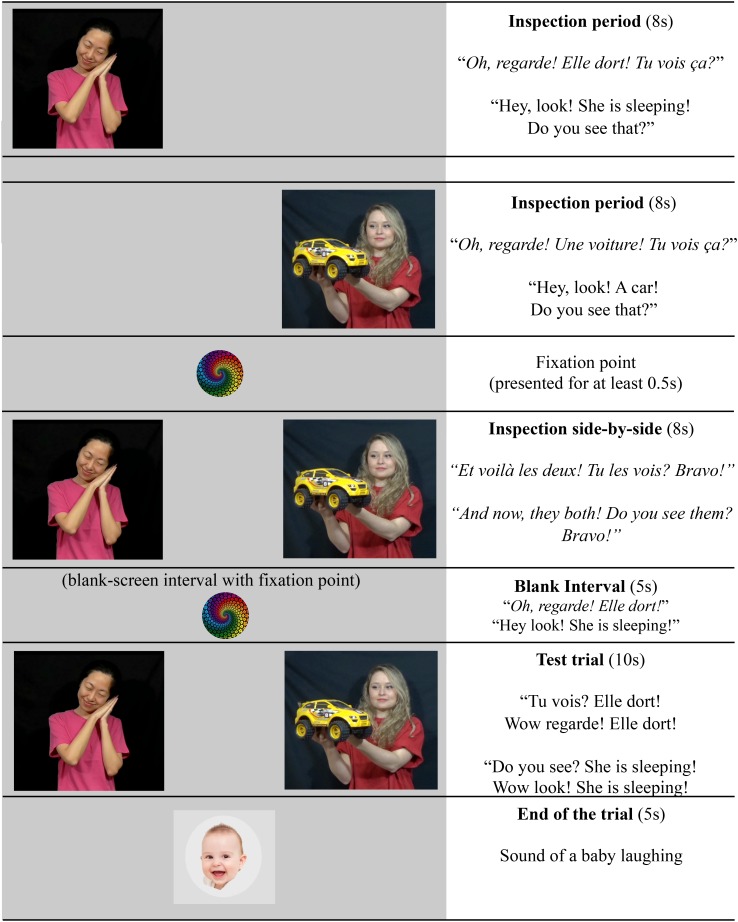
Time-course of the familiar (practice) trials presentation. The novel-word trials were presented in the same way with the exception that during the inspection period the prompt sentences were neutral: they did not contain the novel words and simply asked children to look at the videos (e.g., “Oh look! Do you see that?”).

In order to introduce participants to the task, the procedure began with the two practice trials, involving either a familiar noun or a familiar verb in each trial. Half of the children had “a ball” as the familiar noun trial and “to sleep” as the familiar verb trial. The other half had “a car” as the familiar noun trial and “to eat” as the familiar verb trial. The pairs of videos were identical for all participants. These practice trials served to show children that in this experiment, only one of the two videos matched the soundtrack they heard. Additionally, they allowed us to investigate children’s overall performance in our task, and whether there would be any difference in the processing of familiar words compared to novel words. The time course of the practice trials is illustrated in Figure 3. The side of the target videos (left or right) was counterbalanced across participants.

As illustrated in Figure 3, each trial started with an inspection period during which a video was presented on one side of the TV-screen to provide participants enough time to inspect each of the videos individually (8s for each video). These individual presentations were accompanied by prompt sentences asking children to look at the videos and introducing the familiar target words to them (e.g., “Oh Look! She is sleeping! Do you see that?” or “Oh look! A car! Do you see that?”). Both videos were then simultaneously presented on the screen (8s), 17 cm apart from one another, with a sentence contrasting the two videos and asking participants to look at both videos (e.g., *Et voilà les deux, tu les vois? Bravo!* – “And now look at they both, do you see them? Bravo!”). This inspection side-by-side phase was used to give children the opportunity to see that the two videos would appear together on the screen, rather than surprising them with this simultaneous presentation of both videos at the same time when they were performing the test.

Right after the presentation of both videos together, they disappeared and a colorful fixation target appeared in the middle of the screen while participants heard one exemplar of the test sentence (e.g., “Hey, look! She is sleeping!”), while the screen remained empty for 5s. Next, the two videos reappeared side-by-side on the screen for 10s, and at the same time participants heard the test sentence repeating the target word twice (e.g., Do you see? She is sleeping! Look! She is sleeping!). After the 10s of test, participants saw a picture of a baby in the middle of the screen and heard a sound of a baby laughing.

The four novel-word trials were presented exactly in the same way described for the practice items in Figure 3. The only difference was that during the inspection period, the prompt sentences were neutral and did not contain the novel words: they simply asked children to look at the videos (e.g., “Oh look! Do you see that?”). The side of the test video presentations was counterbalanced within participants, such that for half of the items, a given participant saw the novel action video on the left and the novel object video on the right and for the other half, she had the reverse. The order of presentation of the novel-word items was random.

### Data Processing and Analysis

Before statistical analysis, the data was down-sampled by a factor of 10, by averaging the data from 10 adjacent samples, so that the final sampling rate was one sample every 20 ms. For familiar word trials, thirteen trials out of 194 were removed from the statistical analysis (7 trials from the 20-month-old group, and 6 trials from the 3-to-4-year-old group), because within these trials more than 25% of the data frames were missing between the onset and the end of the trial. After exclusions, each participant in the 20-month-old group contributed an average of 1.85 (*SD* = 0.35) out of 2 familiar-word trials, and each participant in the 3-to-4-year-old group contributed an average of 1.87 (*SD* = 0.33) out of 2 familiar-word trials.

For novel-word trials, sixty-three trials out of 388 were removed from the statistical analysis (37 trials from the 20-month-old group, and 26 trials from the 3-to-4-year-old group), because within these trials more than 25% of the data frames were missing between the onset and the end of the trial. After exclusions, each participant in the 20-month-old group contributed an average of 3.24 (*SD* = 0.82) out of 4 novel-word trials, and each participant in the 3-to-4-year-old group contributed an average of 3.45 (*SD* = 0.71) out of 4 novel-word trials. Given that the looking times toward the action video and toward the object video are almost complementary (apart from the looking away time), we used the proportion of looking times toward the action video as the dependent variable in our statistical analysis. Our prediction was that participants would look more toward the video showing a person performing a novel action when listening to sentences in the verb condition than to sentences in the noun condition.

To find the time-window(s), if any, in which there was a significant difference between conditions, a cluster-based permutation analysis was conducted (similar to: Von Holzen and Mani, 2012; Ferguson et al., 2014, 2018; Dautriche et al., 2015; Hahn et al., 2015; de Carvalho et al., 2016, 2017; Havron et al., 2018; see Maris and Oostenveld, 2007 for a formal presentation of that analysis). This analysis allows us to test for the effect of Condition without inflating the rate of Type I error and has the advantage of allowing us to identify a time-window where we observe a significant effect of condition without having to select it arbitrarily. This analysis is conducted in two steps: (1) the identification of time-windows that have a potential effect; (2) the statistical test itself, which quantifies whether these effects (identified in step 1) are likely to have been generated by chance.

In the first step of this analysis, for each time point, a paired two-tailed *t*-test testing for the effect of Condition (Noun vs. Verb) on the proportion of looks toward the action video was conducted. Adjacent time points with a *t*-value greater than some predefined threshold (here, *t* = 1.5, on arcsin-transformed data) were grouped together into a cluster. The size of the cluster is defined as the sum of the *t*-values at each time point within the cluster.

In the second step of this analysis, to obtain the probability of observing a cluster of that size by chance, 1000 simulations randomly shuffling the conditions (noun, verb) for each trial was conducted. For each simulation, the analysis calculated the size of the biggest cluster identified with the same procedure that was applied to the real data. A cluster of adjacent time points from the real data shows a significant effect of condition if the sum of the *t*-values in this particular cluster was greater than the highest *t*-value sum derived from clusters in 95% of the simulations, which ensures a *p*-value of 0.05. This analysis was conducted on the total duration of each trial (10s), for both the two familiar-word trials (within participants) and the four novel-word trials (between participants). The reason why we decided to conduct the analysis on the entire duration of the test trials rather than only from the onset of the target words was that an effect may exist from the beginning of the test trials since participants could have potentially anticipated their gaze direction after having heard the sentence played one time while the screen was blank, just before the two videos reappeared on the screen. Data analyses and graphics were performed with R software version 3.2.2 (R Core Team, 2015) and the eyetracking R package (Dink and Ferguson, 2015).

## Results

### Familiar Trials

**FIGURE 4:**
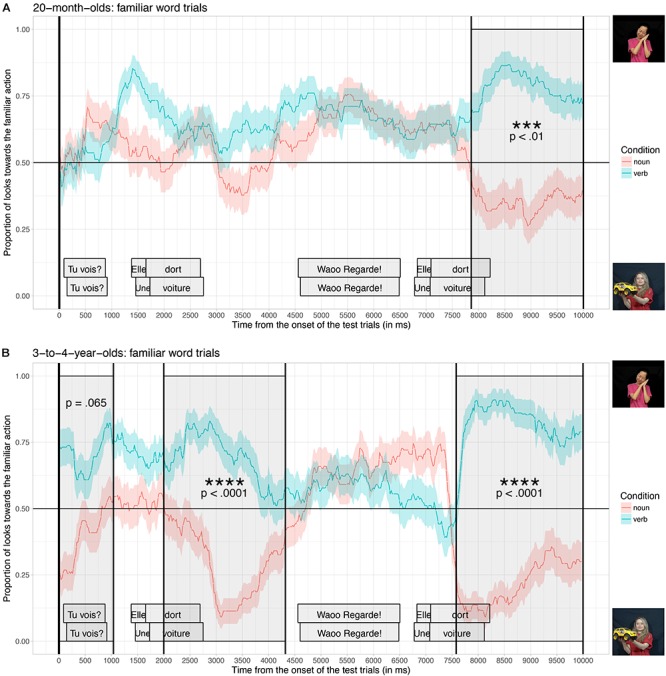
Proportion of looks toward the familiar action, time-locked to the onset of the test trials (vertical black line) for 20-month-olds **(A)**, and 3-to-4-year-olds **(B)**, for children who listened to sentences in the noun condition (red curve) and in the verb condition (blue curve). The cluster-based permutation test revealed significant differences between the noun and the verb conditions (dark gray windows).

Figure 4 shows the proportion of looks toward the video illustrating the familiar actions averaged across the two practice trials, when participants listened to sentences in the verb condition (blue curve, targets: to sleep, to eat) and when they listened to sentences in the noun condition (red curve: targets: ‘a car’ and ‘a ball’), time-locked to the beginning of the test trials, for the 20-month-old group (A) and for the 3-to-4-year-old group (B).

For both age groups, the cluster-based analysis found significant time-windows where the proportion of looks toward the familiar action was significantly different in the verb condition compared to the noun condition. For participants in the 20-month-old group (Figure 4A), only one significant time-window was found. This time-window coincides with the second repetition of the target word during the test (from 7860 ms until 10000 ms, the end of the trial; *p* < 0.01). For participants in the 3-to-4-year-old group (Figure 4B), two significant time-windows were found: the first one coinciding with the first onset of the target word during the test (from 2000 ms until 4320 ms; *p* < 0.0001) and the second one coinciding with the second repetition of the target word during the test (from 7580 ms until 10000 ms, the end of the trial; *p* < 0.0001); a third time-window (from 0 to 1040 ms) was marginally significant (*p* = 0.065).

These results demonstrate that both age groups tended to look more toward the video illustrating the familiar action when they listened to sentences containing a familiar verb than when they listened to sentences containing a familiar noun. When listening to verb sentences, 3-to-4-year-olds increased their looks toward the video illustrating the familiar action, starting from the onset of the test trials, and whenever they heard the target word during the test. The anticipatory looks toward the right video from the very beginning of the test trials (although marginally significant) suggest that 3-to-4-year-olds took advantage of the fact that they had heard the sentence before the videos reappeared (i.e., during the black screen interval) to anticipate their answers. Participants in the 20-month-old group were also able to identify the target videos: toddlers listening to verb sentences increased their looks toward the video illustrating the familiar action. However, this effect of condition was significant only after the second repetition of the target word, although there was a slight tendency in the right direction after the first repetition of the target word. Overall, both age groups were able to correctly interpret the sentences containing familiar nouns and verbs, and looked at the correct videos. 3-to-4-year-olds however seemed to be faster and more accurate than 20-month-olds in this task.

Taking into account the fact that infants usually take between 300 to 500 ms to orient their eye-gaze toward a familiar noun referent (e.g., “a car”), while watching two still pictures of familiar objects side-by-side on a screen (e.g., a car vs. a ball) and listening to simple sentences such as “Where is the car?” (e.g., Swingley and Aslin, 2000; Fernald et al., 2008; Ferguson et al., 2014), the performance of 20-month-olds in our experiment suggests that finding noun and verb referents during the inspection of dynamic scenes with agents and objects takes much more time at that age. The present task is more demanding than the studies using still pictures, because young children were watching two dynamic scenes on the TV-screen at the same time they were processing the sentences. As far as we can tell, this is the first time that the real-time interpretation of familiar nouns and verbs during the inspection of two dynamic scenes was investigated. So we didn’t have a clear hypothesis about how much time it would take for infants to orient their eye-gaze toward nouns and verb referents in this task. It is also possible that our youngest group was slower in constraining their interpretations compared to the older group simply because they found the action video more attractive (a person was doing movements repeatedly) than the novel object video (a person was simply holding an object). All these factors together may have contributed to the speed/performance differences between participants in our task.

Nevertheless, these results show that, despite their speed difference, participants in the 20-month-old group as well as in the 3-to-4-year-old group looked more toward the familiar action video when listening to sentences in the verb condition than in the noun condition.

### Novel-Word Trials

**FIGURE 5:**
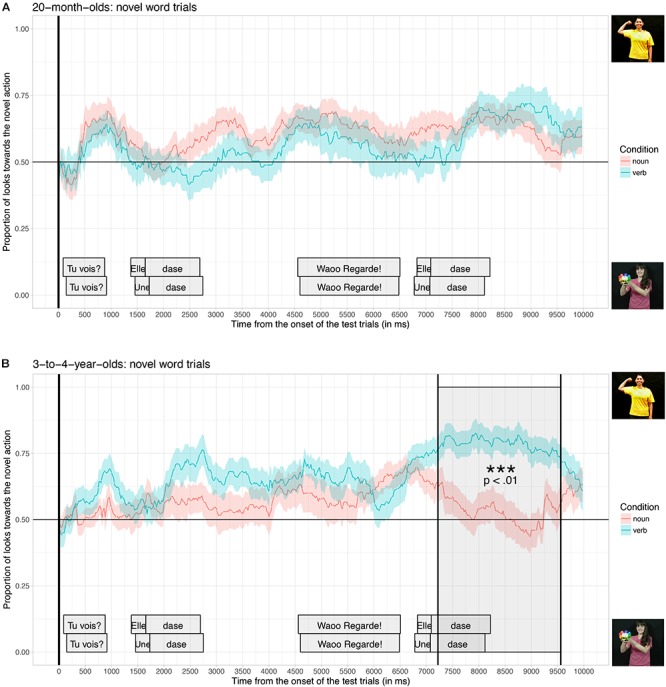
Proportion of looks toward the novel action, time-locked to the onset of the test trials (vertical black line) for 20-month-olds **(A)**, and 3-to-4-year-olds **(B)**, for children who listened to sentences in the noun condition (red curve) and in the verb condition (blue curve). The cluster-based permutation test revealed a significant difference between the noun and the verb conditions (dark gray window) for 3-to-4-year-olds but not for 20-month-olds.

Figure 5 shows the proportion of looks toward the video illustrating the novel actions averaged across the four test trials, when participants listened to sentences in the verb condition (blue curve, e.g., “Look! She is dasing!”) and when they listened to sentences in the noun condition (red curve, e.g., “Look! A dase!”), time-locked to the beginning of the test trials, for the 20-month-old group (A) and for the 3-to-4-year-old group (B).

Visual inspection of the data shows that both groups of children tended to look more toward the video illustrating the novel action than the video showing the novel object, from the beginning of the test trials. However, 3-to-4-year-olds in the verb condition increased their looks toward the novel action, starting slightly after the first onset of the novel target word during the test, and they repeated this behavior even more strongly during the second repetition of the novel target word. Thus, the effect of condition seems to be even stronger around the second repetition of the target word. In contrast, participants in the 20-month-old group did not seem to have behaved differently between the two conditions^2^.

For participants in the 3-to-4-year-old group, the cluster-based analysis found a significant time-window in which the proportion of looks toward the novel action was significantly different in the verb condition compared to the noun condition (from 7220 ms until 9620 ms; *p* = 0.001). This time-window coincides with the second repetition of the novel target word during the test trial (the third occurrence of the novel word). The analysis did not find any significant differences between the two conditions for 20-month-olds.

In order to test this difference between the two age groups statistically, we performed an additional analysis (i.e., an ANOVA) comparing the overall proportion of looking time toward the video illustrating the novel action averaged across the whole trial (10s), across all the four novel words, with participants as the random factor, Condition (Noun vs. Verb) and Age-group (20-month-olds vs. 3-to-4-year-olds) as between-participant factors. This analysis revealed a significant interaction between Condition and Age, *F*(1,93) = 7.825, *p* = 0.006, confirming that the two age groups differ with regard to their sensitivity to the linguistic context. The analysis also confirms, once again, that 3-to-4-year-olds looked more toward the novel action in the Verb condition (Mean = 0.64, *SD*-error = 0.02) than in the noun condition (Mean = 0.53, *SD*-error = 0.03; *F*(1,46) = 8.874, *p* = 0.005) and that this difference was not significant for the 20-month-old group (Mverb = 0.53, *SD*-error = 0.03; Mnoun = 0.56, *SD*-error = 0.02; *F*(1,47) = 0.93, *p* = 0.34).

Taken together, these results show that while participants at both ages were able to associate familiar nouns and familiar verbs to their respective referents presented in dynamic scenes on a TV-screen, only those in the 3-to-4-year-old group were able to rapidly exploit function words to determine the syntactic category of novel words, to infer whether it was more likely to refer to an object (if a noun) or an action (if a verb), and therefore to select the most probable referent to look at. As can be observed in Figure 5B, 3-to-4-year-olds who heard the novel words in the verb condition looked more toward the video depicting a novel action than participants who heard the novel words in the noun condition. Given that participants in the noun and verb condition were exposed to exactly the same videos and target words during the experiment, the only way to explain the difference observed in our results is that young children paid attention to the syntactic context instantiated by function words (a pronoun or a determiner, e.g., *Une dase*! vs. *Elle dase*!) to correctly assign a syntactic category to the novel words and constrain their meanings.

In order to ascertain whether participants who failed to resolve the referents for novel nouns and verbs in our task (i.e., the situation of the 20-month-old group) had successfully identified the referents of familiar nouns and verbs in the practice trials, we conducted an additional analysis using only participants who had the two practice trials (one noun and one verb) with exploitable data. Thus, we excluded all participants who did only one practice trial. We decided to do that simply to make sure that when we compare the performance of participants in the test trials versus the familiar trials, we would be comparing the same participants, rather than only a sub-sample of them in each type of trials in each condition. In this additional analysis we had only 42 participants in each age group, but the same pattern of results was observed. This additional analysis is freely available for readers in the supplementary materials folder on OSF^3^. This additional analysis, together with our current results, confirm that our experiment provides an efficient measure to capture the real-time interpretation of familiar nouns and verbs in 20-month-olds which allow us to conclude that it is not the case that 20-month olds were confused with the experiment and did not do anything. They were simply not able to learn novel word meanings in this experimental paradigm, unlike preschoolers. In other words, 20-month-olds in our study can succeed in an identical experimental setting when they know the meaning of the words, but they might have failed for the novel word trials, because they were unable to infer the meaning of the novel words while listening to only three occurrences of the novel words and watching the two videos side-by-side on the screen.

## Discussion

The study described in this paper shows that with just three occurrences of a novel word in a given syntactic context, 3-to-4-year-olds are able to exploit function words in real-time to determine the syntactic category of novel words and to constrain their possible meanings. In a real-time preferential looking task designed to investigate the time course with which young children can exploit function words to constrain their interpretations of novel nouns and verbs, 3-to-4-year-olds were able to exploit function words in real-time to constrain the possible meaning of novel words, mapping novel nouns to novel objects and novel verbs to novel actions. 20-month-olds, however, did not show this difference when interpreting novel words.

While previous studies investigated young children’s ability to exploit linguistic cues to constrain novel word meanings in situations in which they were first familiarized with repetitive exposures to the linguistic cues and tested after the fact, in the current study children’s learning behavior was measured in real-time during sentence processing. We tested young children’s ability to exploit function words in real-time to determine the syntactic category of novel words and to constrain their meanings while looking at dynamic videos.

In our study, just one or two repetitions of the critical sentences was enough to make young children use syntax as a “zoom lens” to figure out which aspect of the world was being talked about. This finding has important implications for our understanding of which naturally arising uses of words in real-life might be of sufficient quality for learning to take place. Our study suggests that only three occurrences of a novel word in an informative syntactic context might be enough to teach the meaning of novel nouns and verbs to 3-to-4-year-olds, a conclusion that could not be drawn by any of the studies in the literature yet. It also brings evidence that more repetitions might be needed to observe positive effects of learning in 20-month-olds, at least with the present experimental task.

Several factors can be invoked to explain the failure of 20-month-olds to exhibit the expected behavior in this experiment: (1) the cognitive load related to the task itself (i.e., analyzing the content of two videos at the same time they were trying to discover the meaning of the novel words); (2) the rapidity of children’s inferential process (i.e., showing a preference for one of the videos based exclusively on the syntactic computations that they may have done while listening to only three occurrences of the test sentences and watching the videos simultaneously). In other words, it is possible that 20-month-olds’ inferences about novel noun and verb meanings are not yet fast enough: they may need to hear more repetitions of the target word, or simply more time in general, to map the novel word to a possible referent while inspecting dynamic scenes; (3) the fact that we used a simple pronoun or determiner before the target word to provide information about its syntactic category and therefore meaning, rather than using sentences with a more “semantically rich” context to provide syntactic information (as in some of the studies we reviewed in our introduction). In the following paragraphs, we discuss each of these possibilities.

The failure of 20-month-olds to infer novel noun and verb meanings – based on the information provided by function words – in our experiment may seem surprising, given the literature we reviewed in our introduction showing that function words constrain lexical access to familiar words in real-time in children under age two and that at this age infants can make inferences about novel words, depending on the linguistic context in which they appear. However, because we wanted to investigate the speed with which young children can exploit function words to determine the syntactic category of novel words and therefore infer their meanings, our study required us to design a more demanding task for infants than the ones used in previous studies. First, rather than using still pictures (e.g., Ferguson et al., 2014, 2018), we had to use videos to illustrate novel actions and objects. Identifying novel objects and novel actions while watching two videos side-by-side might have been more difficult for young children than inspecting still pictures (which is consistent with the recent findings of Valleau et al., 2018). Consistent with this hypothesis, we also noted that 20-month-olds were slower to interpret even familiar nouns in our task compared to their performance in previous studies using still pictures (e.g., Swingley and Aslin, 2000; Fernald et al., 2008; Ferguson et al., 2014, 2018). This suggests that watching the two videos simultaneously while learning novel word meanings is taxing for young infants, and might have overwhelmed their processing abilities.

Secondly, in previous studies on the acquisition of novel nouns and verbs, children were first taught the meaning of a novel content word while listening to several repetitions of sentences presenting this novel word while they watched only one video at a time (e.g., Bernal et al., 2007; Waxman et al., 2009; He and Lidz, 2017; de Carvalho et al., 2019). In these studies, it was only after the familiarization phase that children were tested on their understanding of the novel word’s meaning: in a preferential looking task, they were given a choice between two videos displayed simultaneously. Thus, the learning of the word meaning was done while only one video was presented on the screen, and the test phase with the two videos side-by-side evaluated which final interpretation children had attributed to the novel word during the familiarization phase. In our study, since we were trying to investigate how these interpretations are constrained in *real-time* from each exposure to the relevant function word, we had to test children at the same time as they were learning the meanings of the novel words. This was a more difficult task because participants were exposed to fewer repetitions of the novel word and visual scenes than in the previous studies (since there was no learning/habituation/dialog phase in our task), and they needed to infer the meaning of the novel word and select the appropriate video, at the same time as they listened to the sentences, which required them to constrain their interpretations in real-time rather than sequentially, after a period of exposure to repetitions of the novel word.

In very recent studies using a less demanding task, 18-month-olds have been shown to be able to exploit function words to determine the syntactic category of novel words and constrain their meanings, after an extensive exposure (i.e., habituation; He and Lidz, 2017; de Carvalho et al., 2019). For instance, using a habituation switch paradigm, these studies first habituated 18-month-olds to several repetitions of a sentence in which a novel word was used as a noun: “Oh look! It’s a *doke*!” as they watched a video showing a penguin doing a spinning action, and to several repetitions of another sentence in which another novel word was used as a verb “Oh look! It’s *pratching*!,” while participants watched another video in which the penguin was doing a cartwheeling action. Then, after having learnt that *doke* means “penguin” and *pratching* means “cartwheeling” during the habituation phase, infants were tested with two trials in which the associations between the sentences and the videos were switched. Infants showed surprise (i.e., looked more to the videos) when listening to verb sentences rendered false by their visual context (“Oh Look! It’s pratching!,” while watching a video showing a penguin spinning); in contrast, they were not surprised when listening to noun sentences that remained true with respect to their visual context, despite the switch (“Oh Look, it’s a doke!” while watching a penguin cartwheeling). This behavior at test was explained by the fact that the kind of switch between the audio tracks of the videos violated the inference constructed about the verb meaning (i.e., “cartwheeling” and “spinning” are different actions), but not about the noun meaning (i.e., although the actions changed, it was always the same penguin in both videos; He and Lidz, 2017; de Carvalho et al., 2019). These studies suggest that when given enough time and provided many repetitions of the novel words in a given syntactic context^4^, even 18-month-olds can use the syntactic context instantiated by function words to make inferences about a novel word meaning. Thus, the failure of 20-month-olds in our task cannot be interpreted as a failure to exploit function words to categorize novel content words and assign meanings to them, but rather as a failure to perform the task when hearing only three occurrences of the novel word and inspecting two videos side-by-side on the screen.

There is evidence in the literature that before age two, toddlers can use function words in real-time to constrain lexical access to known content words (e.g., Kedar et al., 2006, 2017; Cauvet et al., 2014). Several other studies, with offline measures of learning, have also shown that infants can use function words to categorize novel content words from their first birthday (Waxman, 1999; Zangl and Fernald, 2007; Shi and Melançon, 2010; Van Heugten and Johnson, 2011; Cauvet et al., 2014; Haryu and Kajikawa, 2016; He and Lidz, 2017; de Carvalho et al., 2019). What remained unclear from these studies however was (a) whether young children would be able to exploit function words in real-time to determine the syntactic category of novel words and therefore infer their meanings; and (b) how much exposure would be needed for a child to learn from such information. Those were the questions investigated in the current study. We tested whether only three occurrences of a novel word used as either a noun (after a determiner) or as a verb (after a pronoun) would provide enough evidence for a child to exploit its syntactic context and therefore to infer its meaning. Our results show that while both 20-month-olds and 3-to-4-year-olds were able to associate familiar nouns and familiar verbs to their respective referents in real-time while watching two dynamic scenes on a TV-Screen, only 3-to-4-year-olds were able to make inferences about novel word meanings – based on the information provided by function words – when hearing only three repetitions of a novel word in a given syntactic context.

Although the youngest group failed in our task, the success of 3-to-4-year-olds suggests that young children are able to rapidly compute predictions regarding the syntactic category of upcoming and unknown content words based on the information carried by function words. Our results still leave open the possibility that such an efficient mechanism to interpret novel words meanings could also be present at a younger age (although a different experimental design might be necessary to attest it), and may allow young children, in the process of learning their lexicon, to assign a syntactic category to words they have not yet acquired. These results suggest that young children already have the means to retrieve a partial syntactic representation of spoken sentences and attribute a noun or verb meaning to words, depending on the information carried by function words in real-time during sentence processing.

Previous studies with 24-month-olds (Arunachalam and Waxman, 2011, 2015) and also with 3-to-5-year-olds (Imai et al., 2008; Valleau and Arunachalam, 2017) suggested that verb acquisition (contrary to noun acquisition) could be better supported by rich semantic information in the verb’s linguistic context. For instance, in Arunachalam and Waxman (2011), 24- and 27-month-olds easily learned novel nouns when exposed to sentences such as “the girl painted the pilker” (semantically rich context) and/or to sentences such as “she painted the pilker” (sparse^5^ syntactic context). However, to learn novel verb meanings, only participants who were exposed to a novel verb in the semantically rich context succeeded in the task: participants successfully acquired novel verbs in contexts that included full determiner phrases labeling the participants in the event (e.g., “The boy is pilking the balloon”), but they failed to learn the novel verbs in contexts in which the participants in the action were replaced by pronouns (e.g., “He’s pilking it”). The same pattern of results was also observed with older children in Imai et al. (2008) who tested 3-to-5-year-olds and Valleau and Arunachalam (2017) who tested 3-year-olds: more semantically informative contexts (e.g., *The girl is gonna pilk an umbrella*) supported verb acquisition better than “less informative” contexts (e.g., *She is gonna pilk it*). This conclusion with regards to 3-to-5-year-olds is not supported by our current results. Although we did not compare participants’ performance in sentences containing full noun phrases versus sentences containing only subject pronouns, our results demonstrated that 3-to-4-year-olds can also make inferences about novel verb meanings even when the novel verb is embedded in simple “sparse semantic context” and preceded only by a simple pronoun.

However, the results observed with 24-month-olds in these experiments (Arunachalam and Waxman, 2011, 2015) raise the question of whether the failure of 20-month-olds in our experimental task, could be related to the fact that they had to constrain verb interpretations based on a simple pronoun (i.e., sparse syntactic context), rather than with a more semantically informative context (i.e., a full noun phrase before the novel verb). Since we used a different task and provided significantly less exposures to the novel verb and scenes than these previous studies, it remains to be investigated whether 20-month-olds would behave better in our experiment if richer syntactic/semantic information was provided (e.g., “*La fille dase*!” – The girl is dasing; rather than simply using “*Elle dase*” – She’s dasing). Yet, we did not have any reason to believe (before this experiment) that the presence of “pronouns” rather than “full noun phrases” would cause difficulties in novel verb learning for our 20-month-olds. In fact, there is evidence in the literature showing that young children between 18 and 23 months are able to learn novel verbs in sentences containing only pronouns such as “He is gorping” or “It’s pratching” in other experimental designs (Yuan et al., 2012; He and Lidz, 2016, 2017; de Carvalho et al., 2019; Lidz et al., unpublished).

As we mentioned in our introduction, linguistic context is an important mechanism that young children use to constrain the acquisition of novel word meanings (e.g., Gleitman, 1990; Gleitman et al., 2005). The idea behind this hypothesis is that the linguistic context (the syntax) would serve as a “zoom lens” to help listeners focusing their attention on a restricted set of possible referents. In the current study we tested this hypothesis and asked questions about the kind of linguistic information that listeners would exploit to constrain their interpretations (i.e., the role of function words) and the speed with which young children could use this information to constrain their interpretations of novel noun and verb meanings. We directly tested whether the “zoom lens effect” (as originally described by Landau and Gleitman, 1985; Gleitman, 1990; Fisher et al., 1994) could be triggered via function words, and whether this information in turn would impact young children’s visual attention to objects and actions while they simultaneously inspect two dynamic scenes and heard just one to three occurrences of a novel word presented either as a verb (after a pronoun) or as a noun (after a determiner). Our results show that with just three occurrences of a novel word in a given syntactic context, 3-to-4-year-olds are able to exploit function words in real-time to determine the syntactic category of novel words and to constrain their possible meanings, mapping novel nouns to novel objects and novel verbs to novel actions.

Such an ability to interpret speech in real-time might be extremely useful for young children during the first steps of language acquisition. Given that in some instances, when young children hear a sentence containing a word that they don’t know yet, they might not have access to many repetitions of the same word to guess its meaning, the rapid use of linguistic information to focus their attention on the relevant parts of the scene that they are observing might represent an important tool for young children to map words to their possible referents and to boost their acquisition of word meanings. To illustrate this idea, imagine for instance a child who would hear a sentence such as “the boy and the girl are VERB-ing the ball”. We see how having full NP subjects and labeled objects can help children to zoom in on the most probable referent. If within a visual scene listeners have information about who is the agent of the action, this will already severely restricts the place where they are going to focus their attention. For instance, the sentence “the boy is pilking the dog” is assumed to trigger more attention to the action (because the agent and the object are well know) than the sentence “He is pilking it” (because in addition to interpret the novel verb, listeners will also have to figure out who is the agent and the patient of this novel action) (e.g., Arunachalam and Waxman, 2011). So it is possible that young infants simply need more support from the linguistic context to help them focus their attention on the relevant part of the scene when inspecting complex visual scenes. In our case, when learning a novel verb, the pronoun most likely referred to one of the two individuals, but then children still had to choose between the two videos since they saw a person in each video. It is therefore possible that the task would be better performed with some metalinguistic skills, namely judging that one video is a better candidate for an action label than the other, and actively comparing the two possibilities, something that the 3–4-year-olds are better able to do than the 20-month-olds, given our results. It remains to be seen whether 20-month-olds could successfully exploit function words in real-time to infer novel word meanings, in a task that requires less metalinguistic judgment (if such a task can be designed).

In summary, the fact that 20-month-olds did not behave as expected in our experiment does not imply that they are unable to make use of linguistic information to focus their attention on the relevant parts of the scene that they are observing. As we discussed before, the main important difference of our experimental design, in comparison to previous studies, is that we significantly reduced toddlers’ exposure to the novel words and visual scenes prior to the test phase, and we measured their learning behavior in real-time during the preferential looking task, rather than only after several occurrences of the novel word with just one video at a time. Given that infants as young as 18-month-olds can use function words to learn novel word meanings in experiments providing many repetitions of the novel words and visual scenes before the test phase (e.g., He and Lidz, 2017; de Carvalho et al., 2019), we suspect that more repetitions of the novel words and visual scenes might be needed in the present task to observe positive effects of learning with 20-month-olds.

Overall, our findings suggest that during the first years of life, children already possess a powerful mechanism to map words to their possible referents and to boost their acquisition of word meanings. Before 4 years of age, young children become able to successfully exploit function words in real-time to infer the syntactic category of novel words, and this information in turn allows them to guide their interpretations of novel word meanings. When listening to just three occurrences of a novel word in a given syntactic context (after a pronoun or after a determiner), young children can map novel nouns to novel objects and novel verbs to novel events at the same time as they process the sentences. This mechanism might be extremely important during the first stages of language acquisition and it might help infants to constrain the space of possible meanings for words that they do not know yet.

## Author Contributions

AdC, SW, JT, and AC designed the study. AdC and MB performed research. AdC analyzed the data and wrote the manuscript. AC, JT, MB, and SW provided critical revisions.

## Conflict of Interest Statement

The authors declare that the research was conducted in the absence of any commercial or financial relationships that could be construed as a potential conflict of interest.

## Footnotes

1 Going from 6 to 8 repetitions of the critical sentences in each familiarization trial in Waxman et al. (2009), number of repetitions = 6; and in Bernal et al. (2007), number of repetitions = 8. In He and Lidz, 2017 and de Carvalho et al., 2019, a habituation phase prior to the test exposed infants to 16 to 111 repetitions of the critical sentences, depending on how fast the child reached the pre-defined habituation criterion, before starting the test phase.

2 This failure of the 20-month-olds to resolve the referent for novel nouns and verbs in real-time was replicated in our lab with another group of 48 infants (24 in each condition) in a previous version of this same experiment. The same videos and test sentences were used in this replication, the only differences were: (a) the familiar noun and verb referents were not named during the inspection period; (b) there was no inspection side-by-side phase before the test; (c) no fixation points were used; and (d) the laughing baby was not presented at the end of the trials.

3 https://osf.io/wmnvg/?view_only=89ee189843b34f01bc81a14a86396141

4 For instance, in de Carvalho et al. (2019) the habituation phase took about 3 min in average and the sentences were repeated several times until infants became habituated to them (average number of sentences’ repetitions during the habituation phase = 57, *SD* = 24;1, ranging from 16 repetitions to 111 repetitions across participants).

5 That sentence is considered to be sparse because the subject of the action is marked only by a simple pronoun rather than a full noun phrase.
